# Comparative Effectiveness of Licensed Influenza Vaccines in Preventing Influenza-related Medical Encounters and Hospitalizations in the 2022–2023 Influenza Season Among Adults ≥65 Years of Age

**DOI:** 10.1093/cid/ciae375

**Published:** 2024-08-21

**Authors:** Jennifer H Ku, Emily Rayens, Lina S Sy, Lei Qian, Bradley K Ackerson, Yi Luo, Julia E Tubert, Gina S Lee, Punam P Modha, Yoonyoung Park, Tianyu Sun, Evan J Anderson, Hung Fu Tseng

**Affiliations:** Department of Research and Evaluation, Kaiser Permanente Southern California, Pasadena, California, USA; Department of Research and Evaluation, Kaiser Permanente Southern California, Pasadena, California, USA; Department of Research and Evaluation, Kaiser Permanente Southern California, Pasadena, California, USA; Department of Research and Evaluation, Kaiser Permanente Southern California, Pasadena, California, USA; Department of Research and Evaluation, Kaiser Permanente Southern California, Pasadena, California, USA; Department of Research and Evaluation, Kaiser Permanente Southern California, Pasadena, California, USA; Department of Research and Evaluation, Kaiser Permanente Southern California, Pasadena, California, USA; Department of Research and Evaluation, Kaiser Permanente Southern California, Pasadena, California, USA; Department of Research and Evaluation, Kaiser Permanente Southern California, Pasadena, California, USA; Moderna Inc., Cambridge, Massachusetts, USA; Moderna Inc., Cambridge, Massachusetts, USA; Moderna Inc., Cambridge, Massachusetts, USA; Department of Research and Evaluation, Kaiser Permanente Southern California, Pasadena, California, USA; Department of Health Systems Science, Kaiser Permanente Bernard J. Tyson School of Medicine, Pasadena, California, USA

**Keywords:** influenza, influenza vaccine, vaccine effectiveness, epidemiology, influenza-related medical encounters

## Abstract

**Background:**

Influenza causes substantial morbidity, particularly among older individuals. Updated data on the effectiveness of currently licensed vaccines in this population are needed.

**Methods:**

At Kaiser Permanente Southern California, we conducted a retrospective cohort study to evaluate comparative vaccine effectiveness (cVE) of high-dose (HD), adjuvanted, and standard-dose (SD) cell-based influenza vaccines, relative to the SD egg-based vaccine. We included adults aged ≥65 years who received an influenza vaccine between 1 August 2022 and 31 December 2022, with follow-up up to 20 May 2023. Primary outcomes were: (1) influenza-related medical encounters and (2) polymerase chain reaction (PCR)-confirmed influenza-related hospitalization. Adjusted hazard ratios (aHR) were estimated by Cox proportional hazards regression, adjusting for confounders using inverse probability of treatment weighting (IPTW). cVE (%) was calculated as (1—aHR) × 100 when aHR ≤1, and ([1/aHR]—1) × 100 when aHR >1.

**Results:**

Our study population (n = 495 119) was 54.9% female, 46.3% non-Hispanic White, with a median age of 73 years (interquartile range [IQR] 69–79). Characteristics of all groups were well balanced after IPTW. Adjusted cVEs against influenza-related medical encounters in the HD, adjuvanted, and SD cell-based vaccine groups were 9.1% (95% confidence interval [CI]: .9, 16.7), 16.9% (95% CI: 1.7, 29.8), and −6.3 (95% CI: −18.3, 6.9), respectively. Adjusted cVEs against PCR-confirmed hospitalization in the HD, adjuvanted, and SD cell-based groups were 25.1% (95% CI: .2, 43.8), 61.6% (95% CI: 18.1, 82.0), and 26.4% (95% CI: −18.3, 55.7), respectively.

**Conclusions:**

Compared to the SD egg-based vaccine, HD and adjuvanted vaccines conferred additional protection against influenza-related outcomes in the 2022–2023 season in adults ≥65 years. Our results provide real-world evidence of the comparative effectiveness of currently licensed vaccines.

## Teaser

High-dose and adjuvanted influenza vaccines conferred additional protection against influenza-related medical encounters and polymerase chain reaction (PCR)-confirmed influenza-related hospitalization in the 2022–2023 season, compared to standard-dose, egg-based influenza vaccine among adults ≥65 years of age.

Seasonal influenza viruses cause up to 1 billion infections and 3 to 5 million severe illnesses annually worldwide [1], leading to an estimated 650 000 deaths each year [2]. In the United States, an estimated 12 000–52 000 influenza-related deaths occurred between 2010 and 2020 [3]. Although influenza causes substantial morbidity in all age groups [4], a high burden of influenza hospitalization occurs among individuals aged ≥65 years and those with underlying comorbidities [5].

Vaccination represents the best strategy for prevention of influenza, particularly against severe disease, with egg-based vaccines as the historic standard. Besides the diversity and evolution of circulating influenza strains posing challenges to vaccine development [6–9], egg-based manufacturing systems are limited by production capacity, length of production, and egg-adapted viral mutations. Overall, influenza vaccine effectiveness (VE) has remained low (<50% between 2014 and 2022), particularly in seasons of mismatch between the vaccine and circulating strains [10–17]. To improve vaccine platforms and production, high-dose (HD), adjuvanted, recombinant, and standard-dose (SD) cell-based vaccines became available in addition to egg-based SD vaccines [18].

In 2022, the Advisory Committee on Immunization Practices made a preferential recommendation for HD, adjuvanted, and recombinant vaccines over SD vaccines for adults ≥65 years of age [19], based on prior VE studies [20]. Because VE can vary substantially year-to-year, updated, real-world data on the utilization and effectiveness of the currently licensed vaccines, directly compared to the SD egg-based vaccine, are important considering the recent preferential recommendations, as well as to inform current vaccine recommendations and to guide vaccine development. At Kaiser Permanente Southern California (KPSC), we conducted a retrospective cohort study to evaluate comparative vaccine effectiveness (cVE) of HD, adjuvanted, and SD cell-based influenza vaccines, compared to the SD egg-based vaccine, in preventing influenza-related medical encounters and hospitalizations in the 2022–2023 influenza season among adults ≥65 years.

## METHODS

### Study Setting

KPSC is an integrated health care system that serves >4.8 million members with sociodemographic characteristics representative of the diverse Southern California population [21]. KPSC electronic health record (EHR) data comprehensively capture details of patient care, including vaccinations, diagnoses, laboratory tests, and pharmacy records. Care received outside of KPSC is incorporated into the EHR as part of claims reimbursement. Vaccinations received outside of KPSC are imported from external sources, including the California Immunization Registry (CAIR), Epic Systems Care Everywhere, claims (eg, retail pharmacies), and vaccination self-reports by members with valid documentation. This study was approved by the KPSC Institutional Review Board, with a waiver of informed consent.

### Study Population

**Figure 1.**
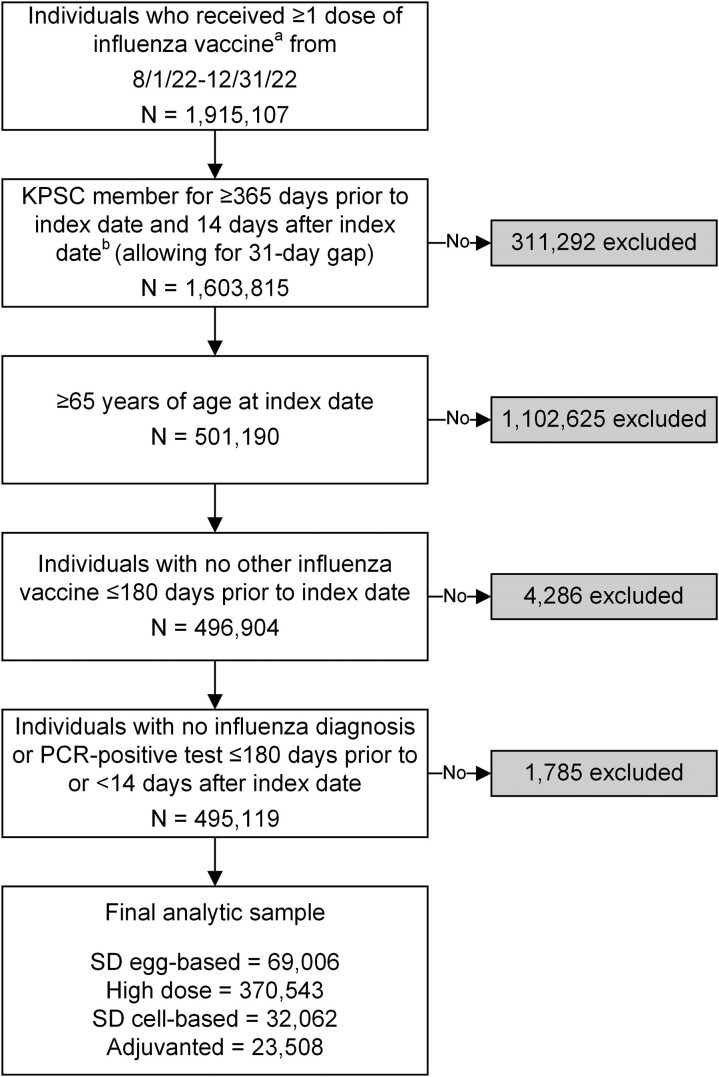
Flow diagram for the analytic cohort. Abbreviations: KPSC, Kaiser Permanente Southern California; PCR, polymerase chain reaction; SD, standard dose. ^a^Influenza vaccines of interest for the exposure included SD egg-based (CVX 150 and 158), high dose (CVX 197), SD cell-based (CVX 171 and 186), and adjuvanted (CVX 205). ^b^Index date was defined as the date of the first dose of influenza vaccine received during the accrual period, based on the CVX codes above. Non-index influenza vaccinations were not limited to the CVX codes above for identifying influenza vaccinations prior to index date (for exclusion criteria and covariates) and after index date (for censoring).

Adults ≥65 years vaccinated with a dose of influenza vaccine during the accrual period (1 August 2022 to 31 December 2022) were eligible; ≥12 months of continuous KPSC membership (allowing for a 31 day gap) prior to and 14 days after the index influenza vaccination date was required (Figure 1). The accrual period considers that the 2022–2023 season was characterized by early influenza activity, starting in October and peaking early December. Index date was defined as the date of the first influenza vaccine received during the accrual period. Individuals were followed from 14 days after their index date until the outcome of interest, death, disenrollment (allowing a 31-day gap), receipt of an additional dose of influenza vaccine, or end of follow-up (20 May 2023), whichever came first. We excluded individuals who: (1) received any other influenza vaccine ≤180 days prior to the index date or (2) had any influenza diagnosis code [22] or influenza polymerase chain reaction (PCR)-positive test ≤180 days prior to or within 14 days following the index date. The event date was the date of the first incident outcome after the index date.

### Exposure and Outcome

The exposure of interest was receipt of a pre-specified licensed influenza vaccine during the accrual period identified using CVX codes (SD egg-based = 150 and 158; HD = 197; adjuvanted = 205; SD cell-based = 171 and 186). Recombinant vaccine, 1 of the 3 preferentially recommended for adults ≥65 years, was not widely administered within KPSC during the study period, and therefore was not included. Individuals who received >1 influenza vaccine during the accrual period were censored upon receipt of the second vaccine. The reference group included recipients of the SD egg-based vaccine as their index vaccination. We identified incident outcomes (first diagnosis during follow-up) through automated EHR search using International Classification of Diseases (ICD)-10 codes and PCR test results. Our 15 hospitals owned and operated by KPSC follow the same guidelines to test admitted patients with acute respiratory infection (ARI) symptoms; testing for influenza was considered complete for members hospitalized with ARI symptoms. The incident outcome date was determined by the earlier of the associated diagnosis date or PCR-positive test date. Primary outcomes were: (1) influenza-related medical encounters (positive influenza PCR test or influenza-related medical encounter in outpatient, inpatient, emergency, or virtual visits identified by influenza [22] ICD-10 codes without an influenza PCR-negative test ±3 days of the code date); and (2) PCR-confirmed influenza-related hospitalization (positive PCR test collected between −14 and +3 days from the inpatient admission date with an ARI code) [23]. Secondary outcomes were identified based on ICD-10 codes: (1) pneumonia hospitalization (ICD-10 J12-J18); and (2) cardiorespiratory hospitalization (ICD-10 I00-I99, J00-J99).

### Covariates

We extracted demographic/clinical covariates from EHR. Variables assessed at index date included age, sex, race/ethnicity, Medicaid status, and household income. Variables assessed in the one or two years prior to the index date included Charlson comorbidity score, frailty index, chronic diseases, respiratory conditions, smoking, body mass index, and health care utilization. Other variables included immunocompromised status, receipt of influenza vaccine during the previous influenza season, concomitant vaccines, and month of index vaccination.

### Statistical Analysis

We described baseline characteristics of the comparison groups (HD, adjuvanted, and SD cell-based) and reference group (SD egg-based). Categorical variables were compared using the chi-square test, and continuous variables were compared using the Kruskal-Wallis test. Absolute standardized differences (ASD) were computed for each pair of groups (HD, adjuvanted, and SD cell-based vs SD egg-based), and the maximum ASD was used to assess the balance of the distribution of covariates. To adjust for potential confounders, an inverse probability of treatment weighting (IPTW) approach was used [24]. First, we used multinomial logistic regression to estimate the probability of receiving a comparison vaccine (ie, propensity score) for each individual, adjusting for baseline characteristics. The weight for each individual was calculated as the inverse of the propensity score and then stabilized by dividing by the average weight for the given exposure group. The distribution of covariates and ASDs were assessed before and after weighting, considering ASD <0.10 a negligible difference.

Unadjusted and adjusted hazard ratios (aHR) comparing incidence rates (IR) of outcomes in each of the comparison groups and the reference group were estimated by Cox proportional hazards regression models without and with weighting, respectively. We performed stratified analyses by age groups (65–74 and ≥75 years), adjusting for any covariates that were no longer balanced after stratifying (ASD >0.10 after weighting). cVE (%) was calculated as (1—aHR) × 100 when aHR ≤1, and ([1/aHR]—1) × 100 when aHR >1. All analyses were conducted using SAS (version 9.4; SAS Institute Inc, Cary, North Carolina, USA).

## RESULTS

**Figure 2.**
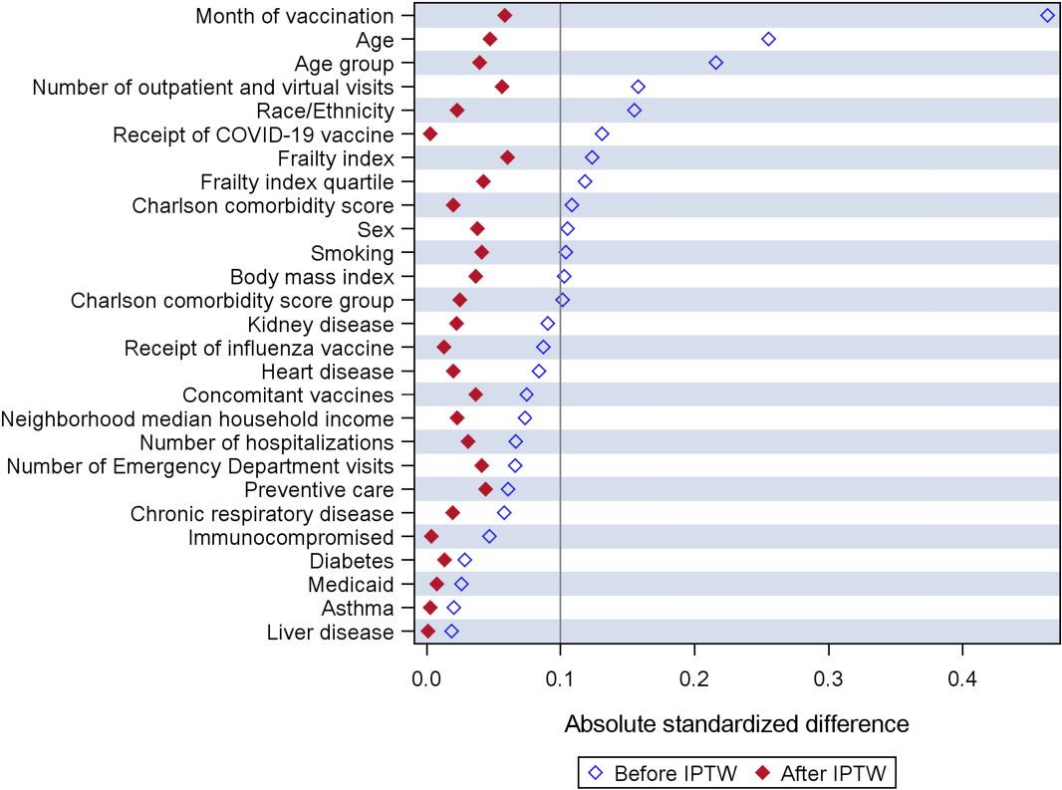
Comparison of absolute standardized difference among influenza vaccine recipients ≥65 y of age, before and after inverse probability of treatment weighting. Abbreviations: COVID-19, coronavirus disease 2019; IPTW, inverse probability of treatment weighting. Chronic respiratory disease includes chronic obstructive pulmonary disease, chronic bronchitis, or emphysema.

Our study included 495 119 adults aged ≥65 years who met the eligibility criteria among the 1 915 107 individuals who received ≥1 dose of influenza vaccine during the accrual period (Figure 1). The overall cohort was 54.9% female and 46.3% non-Hispanic White, with a median age of 73 years (interquartile range [IQR] 69–79). Individuals were followed up for up to 9.2 months (median 7.2 months [IQR 6.4–7.7] for influenza-related medical encounters, and 7.2 months [IQR 6.5–7.7] for PCR-confirmed hospitalization). Among the 495 119 individuals, 69 006 (13.9%) received a SD egg-based vaccine, 370 543 (74.8%) received a HD vaccine, 23 508 (4.7%) received an adjuvanted vaccine, and 32 062 (6.5%) received a SD cell-based vaccine for their index vaccination (Table 1). Individuals who received HD or adjuvanted vaccine tended to be slightly older (mean 74.6 and 75.1 years, respectively) than SD group (73.3 years; Supplementary Table 1). Compared to the other groups, the adjuvanted group also tended to have higher comorbidity burden (mean 2.26 Charlson comorbidity score), was more likely to receive the vaccine earlier (84.2% during August-September 2022) and was more likely to receive a coronavirus disease 2019 (COVID-19) vaccine (90.4%) in the year prior. Baseline demographic and clinical characteristics, as well as health utilization, were well balanced (ASD <0.1) among the comparison and reference groups after IPTW (Figure 2).

**Table 1.** Characteristics of Influenza Vaccine Recipients (≥65 y) by Vaccine Type (After Inverse Probability of Treatment Weighting)

n (%)	Reference Group	Comparison Groups			ASD
	SD Egg-based n = 69 006	High Dose n = 370 543	Adjuvanted n = 23 508	SD Cell-based n = 32 062	
**Demographic characteristics**					
Age at index date, y					0.05
Mean (standard deviation)	74.5 (7.4)	74.4 (7.0)	74.8 (7.3)	74.4 (7.3)	
Median (Q1, Q3)	73 (68, 79)	73 (69, 79)	74 (69, 79)	73 (68, 79)	
min, max	65, 104	65, 110	65, 104	65, 103	
Age at index date, y					0.04
65–74	39 104 (56.7)	211 908 (57.2)	12 861 (54.7)	18 163 (56.7)	
≥75	29 902 (43.3)	158 635 (42.8)	10 647 (45.3)	13 899 (43.3)	
Sex					0.04
Female	38 119 (55.2)	203 474 (54.9)	12 546 (53.4)	17 628 (55.0)	
Male	30 887 (44.8)	167 069 (45.1)	10 962 (46.6)	14 434 (45.0)	
Race/Ethnicity					0.02
Non-Hispanic White	31 983 (46.3)	171 460 (46.3)	10 902 (46.4)	14 873 (46.4)	
Non-Hispanic Black	5852 (8.5)	30 832 (8.3)	2133 (9.1)	2685 (8.4)	
Hispanic	19 735 (28.6)	106 997 (28.9)	6624 (28.2)	9238 (28.8)	
Non-Hispanic Asian	9137 (13.2)	48 937 (13.2)	3055 (13.0)	4230 (13.2)	
Other/Unknown	2299 (3.3)	12 317 (3.3)	794 (3.4)	1036 (3.2)	
Medicaid	4267 (6.2)	22 777 (6.1)	1497 (6.4)	1996 (6.2)	0.01
Neighborhood median household income					0.02
<$40 000	1079 (1.6)	5816 (1.6)	401 (1.7)	510 (1.6)	
$40,000–$59 999	8473 (12.3)	45 892 (12.4)	2830 (12.0)	3983 (12.4)	
$60,000–$79 999	13 772 (20.0)	74 380 (20.1)	4593 (19.5)	6507 (20.3)	
≥$80 000	45 468 (65.9)	243 340 (65.7)	15 590 (66.3)	20 967 (65.4)	
Unknown	213 (0.3)	1116 (0.3)	94 (0.4)	95 (0.3%)	
Smoking^a^					0.04
No	49 330 (71.5)	265 074 (71.5)	16 480 (70.1)	22 940 (71.5)	
Yes	18 017 (26.1)	96 737 (26.1)	6330 (26.9)	8396 (26.2)	
Unknown	1660 (2.4)	8732 (2.4)	698 (3.0)	726 (2.3)	
Clinical characteristics					
Body mass index^a^, kg/m^2^					0.04
<18.5	1281 (1.9)	6552 (1.8)	465 (2.0)	563 (1.8)	
18.5 to < 25	19 504 (28.3)	104 181 (28.1)	6496 (27.6)	9013 (28.1)	
25 to < 30	24 211 (35.1)	130 121 (35.1)	8121 (34.5)	11 252 (35.1)	
≥30	21 149 (30.6)	114 398 (30.9)	7297 (31.0)	9955 (31.0)	
Unknown	2862 (4.1)	15 291 (4.1)	1130 (4.8)	1280 (4.0)	
Charlson comorbidity score^b,c^					0.03
0	21 764 (31.5)	117 411 (31.7)	7352 (31.3)	10 101 (31.5)	
1	13 948 (20.2)	74 930 (20.2)	4558 (19.4)	6501 (20.3)	
≥2	33 293 (48.2)	178 202 (48.1)	11 598 (49.3)	15 460 (48.2)	
Frailty index^b,d^					0.04
Quartile 1	17 186 (24.9)	92 658 (25.0)	5714 (24.3)	7936 (24.8)	
Quartile 2	17 105 (24.8)	92 599 (25.0)	5711 (24.3)	8030 (25.0)	
Quartile 3	17 252 (25.0)	92 595 (25.0)	5699 (24.2)	8059 (25.1)	
Quartile 4, most frail	17 463 (25.3)	92 691 (25.0)	6384 (27.2)	8037 (25.1)	
Chronic diseases^b^					
Kidney disease	13 639 (19.8)	72 796 (19.6)	4855 (20.7)	6351 (19.8)	0.02
Heart disease	7115 (10.3)	37 859 (10.2)	2566 (10.9)	3305 (10.3)	0.02
Liver disease	3495 (5.1)	18 831 (5.1)	1189 (5.1)	1625 (5.1)	<0.01
Diabetes	21 911 (31.8)	117 722 (31.8)	7610 (32.4)	10 232 (31.9)	0.01
Immunocompromised	3842 (5.6)	20 666 (5.6)	1290 (5.5)	1798 (5.6)	<0.01
Respiratory conditions^b^					
Chronic obstructive pulmonary disease, chronic bronchitis, or emphysema	5508 (8.0)	29 354 (7.9%)	2002 (8.5)	2550 (8.0)	0.02
Asthma	6665 (9.7)	35 763 (9.7)	2285 (9.7)	3123 (9.7)	<0.01
**Healthcare utilization**					
Number of outpatient and virtual visits^b^					0.06
0	1204 (1.7)	6236 (1.7)	508 (2.2)	513 (1.6)	
1–4	9982 (14.5)	53 747 (14.5)	3780 (16.1)	4608 (14.4)	
5–10	21 121 (30.6)	114 051 (30.8)	7107 (30.2)	9850 (30.7)	
≥11	36 699 (53.2)	196 509 (53.0)	12 114 (51.5)	17 091 (53.3)	
Number of Emergency Department visits^b^					0.04
0	53 079 (76.9)	285 684 (77.1)	17 718 (75.4)	24 668 (76.9)	
1	10 370 (15.0)	55 392 (14.9)	3664 (15.6)	4829 (15.1)	
≥2	5557 (8.1)	29 467 (8.0)	2126 (9.0)	2565 (8.0)	
Number of hospitalizations^b^					0.03
0	63 342 (91.8)	340 674 (91.9)	21 387 (91.0)	29 467 (91.9)	
1	4050 (5.9)	21 418 (5.8)	1485 (6.3)	1858 (5.8)	
≥2	1614 (2.3)	8451 (2.3)	637 (2.7)	737 (2.3)	
Preventive care^b^	38 668 (56.0)	207 532 (56.0)	12 659 (53.9)	18 011 (56.2)	0.04
History of receipt of influenza vaccine^e^	63 401 (91.9)	340 668 (91.9)	21 516 (91.5)	29 487 (92.0)	0.01
Receipt of COVID-19 vaccine^b^	60 382 (87.5)	324 047 (87.5)	20 554 (87.4)	28 026 (87.4)	<0.01
Concomitant vaccines^f^	15 650 (22.7)	84 714 (22.9)	5694 (24.2)	7202 (22.5)	0.04
Month of index vaccination					0.06
August 2022	4052 (5.9)	21 684 (5.9)	1454 (6.2)	1858 (5.8)	
September 2022	34 000 (49.3)	182 002 (49.1)	12 179 (51.8)	15 946 (49.7)	
October 2022	20 080 (29.1)	108 366 (29.2)	6465 (27.5)	9203 (28.7)	
November 2022	7670 (11.1)	41 220 (11.1)	2416 (10.3)	3555 (11.1)	
December 2022	3204 (4.6)	17 272 (4.7%)	994 (4.2)	1500 (4.7)	

Abbreviations: ASD, absolute standardized difference; COVID-19, coronavirus disease 2019; Q, quartile; SD, standard dose.

^a^Defined in the 2 years prior to index date.

^b^Defined in the 1 year prior to index date.

^c^Possible range: 0–29 [25].

^d^Possible range: 0–1 [26].

^e^During previous influenza season (August 2021– April 2022).

^f^Among individuals who received concomitant vaccines received with the index influenza vaccine: COVID-19 vaccine (40.9%); shingles vaccine (47.9%); pneumococcal vaccine (12.1%); Tdap (9.0%); and other vaccines (0.7%)

(47.9%); pneumococcal vaccine (12.1%); Tdap (9.0%); and other vaccines (0.7%).

The incidence rate (IR) for influenza-related medical encounters per 1000 person-years in the SD egg-based dose group was 15.6 (95% confidence interval [CI]: 14.4, 16.8), whereas IRs in the HD, adjuvanted, and SD cell-based groups were 14.0 (95% CI: 13.5, 14.5), 14.1 (12.2, 16.1), and 17.5 (15.7, 19.5), respectively (Figure 2, Table 2). The IR for PCR-confirmed hospitalization per 1000 person-years in the SD egg-based vaccine group was 1.3 (1.0, 1.7), while IRs in the HD, adjuvanted, and SD cell-based groups were 1.1 (1.0, 1.3), 0.6 (0.3, 1.2), and 1.1 (0.7, 1.6), respectively. The IR for pneumonia hospitalizations was 21.3 (19.9, 22.8) in the SD egg-based group, with point estimates ranging between 22.6 and 28.0 in the HD, adjuvanted, and SD cell-based vaccine groups. The IR for cardiorespiratory hospitalizations was 107.1 (103.9, 110.4) in the SD egg-based group, with point estimates ranging between 109.4 and 128.1 in the HD, adjuvanted, and SD cell-based vaccine groups.

**Table 2.** Incidence Rates and Comparative Vaccine Effectiveness of HD, Adjuvanted, and SD Cell-based Influenza Vaccines in Preventing Influenza/Respiratory Related Outcomes, Compared to SD Egg-based Influenza Vaccine, Among Influenza Vaccine Recipients ≥65 y of age

	Reference Group								Comparison Groups						
	SD Egg-based (n = 69 006)			HD (n = 370 543)				Adjuvanted (n = 23 508)				SD Cell-based (n = 32 062)			
Outcomes	No. cases	No. p-y	Incidence/1000 p-y (95% CI)	No. cases	No. p-y	Incidence/1000 p-y (95% CI)	Adjusted^a^ cVE (%) (95% CI)	No. cases	No. p-y	Incidence/1000 p-y (95% CI)	Adjusted^a^ cVE (%) (95% CI)	No. cases	No. p-y	Incidence/1000 p-y (95% CI)	Adjusted^a^ cVE (%) (95% CI)
Influenza-related medical encounters^b^	625	40 187.4	15.6 (14.4, 16.8)	2932	210 065.1	14.0 (13.5, 14.5)	9.1 (0.9, 16.7)	201	14 296.9	14.1 (12.2, 16.1)	16.9 (1.7, 29.8)	330	18 875.0	17.5 (15.7, 19.5)	−6.3 (−18.3, 6.9)
PCR-confirmed hospitalization^c^	53	40 409.4	1.3 (1.0, 1.7)	234	211 103.7	1.1 (1.0, 1.3)	25.1 (0.2, 43.8)	9	14 372.8	0.6 (0.3, 1.2)	61.6 (18.1, 82.0)	20	18 999.2	1.1 (0.7, 1.6)	26.4 (−18.3, 55.7)
Pneumonia hospitalization	857	40 230.0	21.3 (19.9, 22.8)	5062	210 032.6	24.1 (23.5, 24.8)	−0.9 (−7.6, 5.9)	400	14 286.0	28.0 (25.4, 30.9)	−10.7 (−21.0, 1.0)	427	18 899.0	22.6 (20.6, 24.8)	−8.7 (−18.2, 2.0)
Cardiorespiratory hospitalization	4203	39 245.4	107.1 (103.9, 110.4)	23 758	204 727.6	116.1 (114.6, 117.5)	0.6 (−2.5, 3.8)	1775	13 856.3	128.1 (122.3, 134.2)	−3.1 (−8.5, 2.6)	2015	18 414.0	109.4 (104.8, 114.3)	−1.2 (−6.2, 3.9)

Abbreviations: CI, confidence interval; cVE, comparative vaccine effectiveness; HD, high dose; no., number; PCR, polymerase chain reaction; p-y, person-years; SD, standard dose.

When the hazard ratio or its 95% CI was >1, the cVE (%) or its 95% CI was transformed as ([1/hazard ratio]—1) × 100.

^a^Weighted using stabilized inverse probability of treatment weights.

^b^A positive influenza PCR test; or influenza-related medical encounter in the outpatient, inpatient, emergency, or virtual visit setting identified based on the US Armed Forces Health Surveillance Center Code Set B [22] for influenza without an influenza PCR-negative test ±3 d of the code date.

^c^PCR-confirmed influenza-related hospitalization (a positive PCR test collected between −14 and +3 d from the inpatient admission date) with an acute respiratory infection code [23].

**Figure 3.**
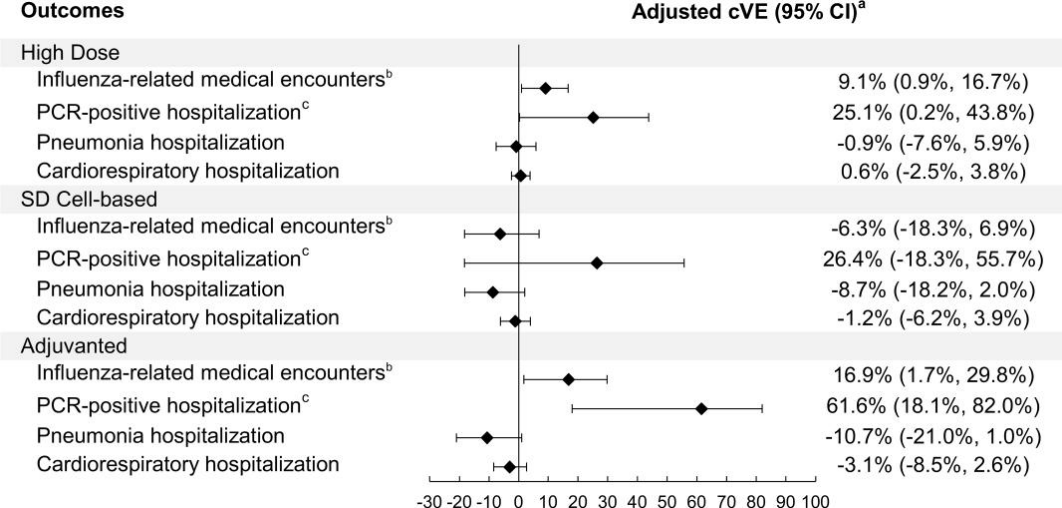
Forest plot: adjusted cVE for high-dose, adjuvanted, and SD cell-based influenza vaccines, compared to SD egg-based vaccine. Abbreviations: CI, confidence interval; cVE, comparative vaccine effectiveness (vs SD egg-based); PCR, polymerase chain reaction; SD, standard dose. When the hazard ratio or its 95% CI was >1, the cVE (%) or its 95% CI was transformed as ([1/hazard ratio]—1) × 100. ^a^Weighted using stabilized inverse probability of treatment weights. ^b^A positive influenza PCR test; or influenza-related medical encounter in the outpatient, inpatient, emergency, or virtual visit setting identified based on the US Armed Forces Health Surveillance Center Code Set B [22] for influenza without an influenza PCR-negative test ±3 d of the code date. ^c^PCR-confirmed influenza-related hospitalization (a positive PCR test collected between −14 and +3 d from the inpatient admission date) with an acute respiratory infection code [23].

Adjusted cVEs against influenza-related medical encounters in the HD and adjuvanted vaccine groups were 9.1% (0.9, 16.7) and 16.9% (1.7, 29.8), respectively, compared to the SD egg-based group, with non-statistically significant cVE in the SD cell-based vaccine group (−6.3% [−18.3, 6.9]) (Figure 3). Adjusted cVEs against PCR-confirmed hospitalization in the HD and adjuvanted vaccine groups were 25.1% (0.2, 43.8) and 61.6% (18.1, 82.0), respectively, compared to the SD egg-based group, while the adjusted cVE in the SD cell-based group (26.4% [−18.3, 55.7]) was not statistically significant. Adjusted cVEs against pneumonia hospitalization and cardiorespiratory hospitalization were not statistically significant, with point estimates ranging from −10.7% to −0.9%, and from −3.1% to 0.6%, respectively. Results for the stratified analysis by age groups were consistent with the main results, but mostly not statistically significant due to limited sample size (Supplementary Table 2).

## DISCUSSION

We evaluated cVE of currently licensed vaccines (HD, adjuvanted, SD cell-based), using SD egg-based vaccine as the reference group. The preferentially recommended HD and adjuvanted influenza vaccines conferred additional protection for older individuals against influenza-related medical encounters and PCR-confirmed influenza hospitalizations; cVEs were insignificant for SD cell-based vaccine, which is not preferentially recommended in this age group. cVEs remained insignificant and low for all vaccine types against pneumonia hospitalization and cardiorespiratory hospitalization.

Previous studies have compared different types of influenza vaccines to one another in older individuals. Data from immunogenicity studies comparing trivalent SD egg-based vaccine to trivalent HD vaccine in US adults aged ≥65 years demonstrated higher antibody levels with HD vaccine [27]. In a randomized efficacy trial, trivalent HD vaccine induced significantly higher antibody responses and was 24% more efficacious in preventing laboratory-confirmed influenza than SD egg-based vaccine [28]. Similarly, a retrospective cohort study using Medicare data showed significantly higher VE for HD vaccine compared to SD vaccine against influenza-related medical encounters [29]. Conversely, cVE in other observational studies were mixed and varied across seasons, possibly due to differences in dominant strain and/or mismatch between vaccine and circulating strains. In a large US cohort of veterans aged ≥65 years in the 2010–2011 influenza season, HD vaccine was not associated with lower rates of hospitalization for influenza, all-cause hospitalization, pneumonia, or death, compared to SD vaccine, although the risk of hospitalization was lower among recipients aged ≥85 years [30]. A systematic review reported a pooled SD cell-based vaccine cVE of 9.9% (95% CI: 6.9, 12.9) compared to SD egg-based vaccine against influenza-related medical encounters for adults ≥65 years during 2017–2018 season; there was no significant difference in 2018–2019 [31]. In a large US cohort of adults aged ≥65 years using EHR data, adjuvanted trivalent influenza vaccine was shown to provide better protection than SD egg-based quadrivalent vaccine (cVE 18.2% [95% CI: 15.8, 20.5] and 27.0% [95% CI: 24.9, 29.0] against influenza-related medical encounters during the 2017–2018 and 2018–2019 seasons, respectively) [32]. A more recent large US study of older adults (≥65 years) reported cVE of 13.4% (95% CI: 5.1, 21.1) against influenza diagnosis at hospital admission for adjuvanted trivalent influenza vaccine in the 2019–2020 influenza season, compared to SD egg-based vaccine. Furthermore, a study in Medicare beneficiaries (≥65 years) during the 2019–2020 season reported a cVE of 13.3% (95% CI: 7.4, 18.9) for recombinant vaccine, 8.2% (95% CI: 4.2, 12.0) for adjuvanted vaccine, and 6.8% (95% CI: 3.3, 10.1) for HD vaccine against influenza-related hospital encounters, compared to SD egg-based vaccine [33]. These findings were similar to our results; HD and adjuvanted vaccines conferred better protection against influenza-related outcomes. Adjuvants improve influenza VE in several ways, including boosting broad immunogenicity and quality of immune responses to antigens and improving responses in individuals with poor immune function [34]. In our study, the point estimate of cVE for adjuvanted vaccine against PCR-confirmed influenza hospitalization was large (61.6%) with a wide 95% CI (18.1, 82.0). This is most likely due to the relatively small size of the adjuvanted group (4.7% of the study population) in the setting of a rare outcome (IR 0.63 [0.33, 1.20]). Additionally, the low and negative point estimates for cVE of pneumonia hospitalization and cardiorespiratory hospitalization outcomes could partially be explained by non-specific outcomes, as pneumonia and cardiorespiratory hospitalizations could occur due to reasons other than influenza. Other potential explanations include random variation and residual confounding, although these were likely minimal given the well-balanced distribution of covariates after weighting. It is difficult to draw meaningful conclusions from the negative cVE estimates as they were not statistically significant. For interpretation of our results, it is also important to note that influenza type/subtype is a major determinant of disease severity and VE in adults ≥65 years [35], and influenza A(H3N2) was the predominant virus during the 2022–2023 influenza season [36].

Our study has several notable strengths. First, we conducted a large, population-based, real-world study in an integrated health care system with a large, diverse, and stable population. KPSC's comprehensive EHR enabled accurate capture of influenza vaccine exposures, influenza-related outcomes, and other demographic/clinical covariates. Second, we used a common reference group (SD egg-based vaccine) to evaluate the cVE of two licensed enhanced influenza vaccines (HD and adjuvanted) and SD cell-based vaccine. Instead of weighting comparison groups to the reference group separately, we used a generalized logistic regression to estimate propensity scores and weighted each group to the target population (ie, those who received any influenza vaccine). This allowed us to estimate the added protection of each enhanced vaccine in direct comparison with the SD egg-based vaccine from a sample that is generalizable to older adults receiving any influenza vaccine. All individuals included in the study were vaccinated, which minimized confounding by indication which could occur when comparing vaccinated with unvaccinated individuals. Third, additional steps were taken to maximize the validity of outcome definitions. We used PCR results to increase the specificity of our influenza hospitalization outcome definition beyond identification by ARI code. For influenza-related medical encounters, we used a combination of PCR tests and well-validated, highly specific influenza ICD-10 codes [22, 23, 37]. Additionally, any outcome misclassification was likely non-differential between the comparison groups and would not have a substantial impact on the cVE estimates. Fourth, to the best of our knowledge, this study represents the first efforts to evaluate cVE after the US Advisory Committee on Immunization Practices made preferential recommendations for HD, adjuvanted, and recombinant vaccines over SD vaccines for adults ≥65 years of age in 2022 [19]. Lastly, because our study period included follow-up from August 2022 through May 2023, our study included the most relevant data for the 2022–2023 influenza season.

Nonetheless, our study has limitations. First, some influenza vaccines received outside of the KPSC system could be missed. However, KPSC members are incentivized to receive no-cost vaccines within the KPSC system, and external vaccinations are captured in the KPSC EHR data by manual entry with appropriate documentation and electronic updates from CAIR to which providers are required by law to report influenza vaccinations within 24 hours of administration. Therefore, the number of influenza vaccines received outside of KPSC that are not captured in the study should be minimal. Additionally, the distribution of missing outside vaccines is likely non-differential between the comparison and reference groups. Second, outcome misclassification is theoretically possible due to imperfect capture of test results and sensitivity of molecular tests to detect influenza virus. However, molecular influenza diagnostic tests are highly sensitive (90%–95%) [38] and bias from this misclassification is likely minimal. Some individuals hospitalized with influenza may have also been missed due to variability in PCR sensitivity relative to symptom onset [39]. However, detectable viral load has been shown to decline more slowly in patients requiring hospitalization [39], relative to community-tested individuals. Furthermore, the distribution of false negative PCR results should be similar across the comparison groups. Third, although our comparison and reference groups were well balanced after IPTW, there could be unmeasured confounding (eg masking, distancing, handwashing, congregate settings). Fourth, given the 2022 preferential recommendations for individuals ≥65 years [19], vaccine uptake may have been higher for the enhanced influenza vaccines compared to the SD vaccines among high-risk individuals. However, because the characteristics of individuals of all groups were well balanced after weighting, we do not believe that this would have caused considerable bias. Importantly, type of vaccine administered was not based on patient choice but rather on availability and supply of vaccines. Lastly, because this study was conducted at a single US health care system, the findings might not be generalizable to other settings or to uninsured populations. However, demographic characteristics of KPSC members are similar to the sociodemographically-diverse source population [40].

In summary, we observed that the preferentially recommended HD and adjuvanted influenza vaccines conferred additional protection for older adults against influenza-related medical encounters and PCR-confirmed influenza hospitalizations, compared to SD egg-based vaccine, although cVE of SD cell-based vaccine was not statistically significant. cVE remained low and not statistically significant for pneumonia hospitalization and cardiorespiratory hospitalization outcomes. Our results provide up-to-date, real-world evidence of the effectiveness of the currently licensed vaccines, which is important for continued evaluations of existing vaccines and development of new vaccines. Ongoing evaluation and monitoring of effectiveness using real-world evidence over multiple influenza seasons are needed to address the limitations of current influenza vaccines, and to guide manufacturers, regulators, and public health decision-makers in advancing the development and evaluation of new and improved vaccines.

## Supplementary Data

Supplementary materials are available at *Clinical Infectious Diseases* online. Consisting of data provided by the authors to benefit the reader, the posted materials are not copyedited and are the sole responsibility of the authors, so questions or comments should be addressed to the corresponding author.

## Supplementary Material

ciae375_Supplementary_Data

## Notes

***Author Contributions.*** J. H. K., L. S. S., L. Q., B. K. A., Y. P., T. S., E. J. A., and H. F. T. conceived and designed the study. J. H. K., E. R., L. S. S., L. Q., B. K. A., Y. L., J. E. T., G. S. L., P. P. M., Y. P., T. S., E. J. A., and H. F. T. were involved in acquisition, analysis, or interpretation of data. J. H. K. developed the first draft of the manuscript. E. R., L. S. S., L. Q., B. K. A., Y. L., J. E. T., G. S. L., P. P. M., Y. P., T. S., E. J. A , and H. F. T. were involved in the critical revision of the manuscript for important intellectual content. L. Q., Y. L., and J. E. T. performed the statistical analysis. Y. P. and H. F. T. obtained funding for the study. G. S. L., P. P. M., L. S. S., and Y. P. provided administrative, technical, or material support. Y. P., E. J. A., and H. F. T. provided project supervision. All authors contributed to the writing of the manuscript and approved the final version for publication.

***Acknowledgment.*** The authors thank the patients of Kaiser Permanente for their partnership with us to improve their health. Their information, collected through our electronic health record systems, leads to findings that help us improve care for our members and can be shared with the larger community.

***Data sharing statement.*** Individual-level data reported in this study involving human research participants are not publicly shared due to potentially identifying or sensitive patient information. Upon request to corresponding authors [J. H. K. and E. R.]., and subject to review and approval of an analysis proposal, KPSC may provide the deidentified aggregate-level data that support the findings of this study within 6 months. Anonymized data (deidentified data including participant data as applicable) that support the findings of this study may be made available from the investigative team in the following conditions: (1) agreement to collaborate with the study team on all publications, (2) provision of external funding for administrative and investigator time necessary for this collaboration, (3) demonstration that the external investigative team is qualified and has documented evidence of training for human subjects protections, and (4) agreement to abide by the terms outlined in data use agreements between institutions.

***Financial support*.** This work was supported by Moderna, Inc.

## References

[1] World Health Organization . Vaccines against influenza: WHO position paper—may 2022. Wkly Epidemiol Rec 2022; 97:186.

[2] Iuliano AD, Roguski KM, Chang HH, et al Estimates of global seasonal influenza-associated respiratory mortality: a modelling study. Lancet 2018; 391:1285–300. 29248255 10.1016/S0140-6736(17)33293-2 PMC5935243

[3] Centers for Disease Control and Prevention . Influenza: disease burden of flu. Updated 30 November 2023. Available at: https://www.cdc.gov/flu/about/burden/index.html. Accessed 7 January 2024

[4] Centers for Disease Control and Prevention . Estimated flu-related illnesses, medical visits, hospitalizations, and deaths in the United States—2017–2018 flu season. Available at: https://archive.cdc.gov/#/details?url=https://www.cdc.gov/flu/about/burden/2017-2018.htm. Accessed 18 July 2023.

[5] Near AM, Tse J, Young-Xu Y, Hong DK, Reyes CM. Burden of influenza hospitalization among high-risk groups in the United States. BMC Health Serv Res 2022; 22:1209. 36171601 10.1186/s12913-022-08586-y PMC9520810

[6] Nachbagauer R, Palese P. Is a universal influenza virus vaccine possible? Annu Rev Med 2020; 71:315–27. 31600454 10.1146/annurev-med-120617-041310

[7] Monto AS . Reflections on the global influenza surveillance and response system (GISRS) at 65 years: an expanding framework for influenza detection, prevention and control. Influenza Other Respir Viruses 2018; 12:10–2. 29460424 10.1111/irv.12511 PMC5818347

[8] Weir JP, Gruber MF. An overview of the regulation of influenza vaccines in the United States. Influenza Other Respir Viruses 2016; 10:354–60. 27426005 10.1111/irv.12383 PMC4947948

[9] Zost SJ, Parkhouse K, Gumina ME, et al Contemporary H3N2 influenza viruses have a glycosylation site that alters binding of antibodies elicited by egg-adapted vaccine strains. Proc Natl Acad Sci U S A 2017; 114:12578–83. 29109276 10.1073/pnas.1712377114 PMC5703309

[10] Centers for Disease Control and Prevention . CDC seasonal flu vaccine effectiveness studies. Updated February 29, 2024. Available at: https://www.cdc.gov/flu/vaccines-work/effectiveness-studies.htm. Accessed 31 March 2024.

[11] Price AM, Flannery B, Talbot HK, et al Influenza vaccine effectiveness against influenza A(H3N2)-related illness in the United States during the 2021–2022 influenza season. Clin Infect Dis 2023; 76:1358–63. 36504336 10.1093/cid/ciac941 PMC10893961

[12] Tenforde MW, Kondor RJG, Chung JR, et al Effect of antigenic drift on influenza vaccine effectiveness in the United States-2019–2020. Clin Infect Dis 2021; 73:e4244–50. 33367650 10.1093/cid/ciaa1884 PMC8664438

[13] Flannery B, Kondor RJG, Chung JR, et al Spread of antigenically drifted influenza A(H3N2) viruses and vaccine effectiveness in the United States during the 2018–2019 season. J Infect Dis 2020; 221:8–15. 31665373 10.1093/infdis/jiz543 PMC7325528

[14] Zimmerman RK, Nowalk MP, Chung J, et al 2014–2015 influenza vaccine effectiveness in the United States by vaccine type. Clin Infect Dis 2016; 63:1564–73. 27702768 10.1093/cid/ciw635 PMC5146719

[15] Rolfes MA, Flannery B, Chung JR, et al Effects of influenza vaccination in the United States during the 2017–2018 influenza season. Clin Infect Dis 2019; 69:1845–53. 30715278 10.1093/cid/ciz075 PMC7188082

[16] Jackson ML, Chung JR, Jackson LA, et al Influenza vaccine effectiveness in the United States during the 2015–2016 season. N Engl J Med 2017; 377:534–43. 28792867 10.1056/NEJMoa1700153 PMC5727917

[17] Flannery B, Chung JR, Monto AS, et al Influenza vaccine effectiveness in the United States during the 2016–2017 season. Clin Infect Dis 2019; 68:1798–806. 30204854 10.1093/cid/ciy775 PMC6522684

[18] Centers for Disease Control and Prevention. Seasonal Flu Vaccines , Available at: https://www.cdc.gov/flu/prevent/flushot.htm. Accessed 18 July 2023

[19] Grohskopf LA, Blanton LH, Ferdinands JM, et al Prevention and control of seasonal influenza with vaccines: recommendations of the advisory committee on immunization practices—united States, 2022–23 influenza season. MMWR Recomm Rep 2022; 71:1–28. 10.15585/mmwr.rr7101a1 PMC9429824 36006864

[20] Centers for Disease Control and Prevention . CDC Seasonal Flu Vaccine Effectiveness Studies, Available at: https://www.cdc.gov/flu/vaccines-work/effectiveness-studies.htm. Accessed 18 July 2023

[21] Koebnick C, Langer-Gould AM, Gould MK, et al Sociodemographic characteristics of members of a large, integrated health care system: comparison with US census bureau data. Perm J 2012; 16:37–41. 10.7812/tpp/12-031 PMC3442759 23012597

[22] Armed Forces Health Surveillance Center . INFLUENZA-LIKE ILLNESS (ILI) Code Sets: Code Set B. Updated 2015 October, Available at: https://www.health.mil/Reference-Center/Publications/2015/10/01/Influenza-Like-Illness. Accessed 18 July 2023

[23] Tenforde MW, Weber ZA, DeSilva MB, et al Vaccine effectiveness against influenza-associated urgent care, emergency department, and hospital encounters during the 2021–2022 season, VISION network. J Infect Dis 2023; 228:185–95. 36683410 10.1093/infdis/jiad015 PMC11306092

[24] Tartof SY, Qian L, Rieg GK, et al Safety of seasonal influenza vaccination in hospitalized surgical patients: a cohort study. Ann Intern Med 2016; 164:593–9. 26974053 10.7326/M15-1667

[25] Quan H, Li B, Couris CM, et al Updating and validating the Charlson comorbidity index and score for risk adjustment in hospital discharge abstracts using data from 6 countries. Am J Epidemiol 2011; 173:676–82. 21330339 10.1093/aje/kwq433

[26] Kim DH, Schneeweiss S, Glynn RJ, Lipsitz LA, Rockwood K, Avorn J. Measuring frailty in medicare data: development and validation of a claims-based frailty Index. J Gerontol A Biol Sci Med Sci 2018; 73:980–7. doi:10.1093/gerona/glx229 29244057 PMC6001883

[27] Centers for Disease Control and Prevention . High-Dose Flu Vaccine. January 7th, 2024, Updated May 30th, 2023. Available at: https://www.cdc.gov/flu/prevent/qa_fluzone.htm

[28] DiazGranados CA, Dunning AJ, Kimmel M, et al Efficacy of high-dose versus standard-dose influenza vaccine in older adults. N Engl J Med 2014; 371:635–45. 25119609 10.1056/NEJMoa1315727

[29] Izurieta HS, Thadani N, Shay DK, et al Comparative effectiveness of high-dose versus standard-dose influenza vaccines in US residents aged 65 years and older from 2012 to 2013 using medicare data: a retrospective cohort analysis. Lancet Infect Dis 2015; 15:293–300. 25672568 10.1016/S1473-3099(14)71087-4 PMC4834448

[30] Richardson DM, Medvedeva EL, Roberts CB, Linkin DR; Centers for Disease C; Prevention Epicenter P. Comparative effectiveness of high-dose versus standard-dose influenza vaccination in community-dwelling veterans. Clin Infect Dis 2015; 61:171–6. 25829001 10.1093/cid/civ261

[31] Coleman BL, Gutmanis I, McGovern I, Haag M. Effectiveness of cell-based quadrivalent seasonal influenza vaccine: a systematic review and meta-analysis. Vaccines (Basel) 2023; 11:1607. 37897009 10.3390/vaccines11101607 PMC10610589

[32] Boikos C, Fischer L, O'Brien D, Vasey J, Sylvester GC, Mansi JA. Relative effectiveness of adjuvanted trivalent inactivated influenza vaccine versus egg-derived quadrivalent inactivated influenza vaccines and high-dose trivalent influenza vaccine in preventing influenza-related medical encounters in US adults ≥ 65 years during the 2017–2018 and 2018–2019 influenza seasons. Clin Infect Dis 2021; 73:816–23. 33605977 10.1093/cid/ciab152 PMC8423477

[33] Izurieta HS, Lu M, Kelman J, et al Comparative effectiveness of influenza vaccines among US medicare beneficiaries ages 65 years and older during the 2019–2020 season. Clin Infect Dis 2021; 73:e4251–9. 33211809 10.1093/cid/ciaa1727

[34] Tregoning JS, Russell RF, Kinnear E. Adjuvanted influenza vaccines. Hum Vaccin Immunother 2018; 14:550–64. 29232151 10.1080/21645515.2017.1415684 PMC5861793

[35] Langer J, Welch VL, Moran MM, et al High clinical burden of influenza disease in adults aged >/= 65 years: can we do better? A systematic literature review. Adv Ther 2023; 40:1601–27. 36790682 10.1007/s12325-023-02432-1 PMC9930064

[36] Centers for Disease Control and Prevention . Influenza activity in the United States during the 2022–23 season and composition of the 2023–24 influenza vaccine. Updated September 28, 2023. Available at: https://www.cdc.gov/flu/spotlights/2023-2024/22-23-summary-technical-report.htm. Accessed 21 March 2024.

[37] Boikos C, Sylvester GC, Sampalis JS, Mansi JA. Relative effectiveness of the cell-cultured quadrivalent influenza vaccine compared to standard, egg-derived quadrivalent influenza vaccines in preventing influenza-like illness in 2017–2018. Clin Infect Dis 2020; 71:e665–71. 32253431 10.1093/cid/ciaa371 PMC7745007

[38] Centers for Disease Control and Prevention . Overview of Influenza Testing Methods: rapid molecular assays. Updated August 31, 2020. Available at: https://www.cdc.gov/flu/professionals/diagnosis/overview-testing-methods.htm. Accessed 18 July 2023.

[39] Jiang L, Lee VJ, Cui L, et al Detection of viral respiratory pathogens in mild and severe acute respiratory infections in Singapore. Sci Rep 2017; 7:42963. 28218288 10.1038/srep42963 PMC5317157

[40] Davis AC, Voelkel JL, Remmers CL, Adams JL, McGlynn EA. Comparing Kaiser permanente members to the general population: implications for generalizability of research. Perm J 2023; 27:87–98 37170584 10.7812/TPP/22.172 PMC10266863

