# Supplementary material for: Comparative Effectiveness of Licensed Influenza Vaccines in Preventing Influenza-related Medical Encounters and Hospitalizations in the 2022–2023 Influenza Season Among Adults ≥65 Years of Age
Source: Clin Infect Dis. 2024 Aug 21;79(5):1283–92. doi: 10.1093/cid/ciae375 (PMC11581696; doi:10.1093/cid/ciae375)
Supplement: ciae375_Supplementary_Data [file ciae375_supplementary_data.docx]

**Comparative effectiveness of licensed influenza vaccines in preventing influenza-related medical encounters and hospitalizations in the 2022-2023 influenza season among adults ≥65 years of age**

**SUPPLEMENTARY MATERIAL**

Jennifer H. Ku,^1*^ Emily Rayens,^1*^ Lina S. Sy,^1^ Lei Qian,^1^ Bradley K. Ackerson,^1^ Yi Luo,^1^ Julia E. Tubert,^1^ Gina S. Lee,^1^ Punam P. Modha,^1^ Yoonyoung Park,^2^ Tianyu Sun,^2^ Evan J. Anderson,^2^ Hung Fu Tseng^1,3^

^1^Department of Research and Evaluation, Kaiser Permanente Southern California, Pasadena, CA 91101, USA
^2^Moderna Inc., Cambridge, MA 02139, USA

^3^Department of Health Systems Science, Kaiser Permanente Bernard J. Tyson School of Medicine, Pasadena, CA 91101, USA

*Jennifer H. Ku and Emily Rayens contributed equally to this manuscript.

**Corresponding Author:**

Jennifer H. Ku, PhD MPH

Kaiser Permanente Southern California, 100 S Los Robles Ave., Pasadena, CA 91101

Phone: (626) 564-3464

Email: [Jen.H.Ku@kp.org](mailto:Jen.H.Ku@kp.org)

ORCID: 0000-0001-9469-5875

**Co-corresponding author:**

Emily Rayens, PhD MPH

Kaiser Permanente Southern California, 100 S Los Robles Ave., Pasadena, CA 91101

Phone: (626) 564-3625

Email: [Emily.X.Rayens@kp.org](mailto:Emily.X.Rayens@kp.org)

ORCID: 0000-0003-3369-3475

Table of Contents

[**Supplementary Table 1.** Characteristics of influenza vaccine recipients (≥65 years) by vaccine type (before inverse probability of treatment weighting) 3](#_Toc168923701)

[**Supplementary Table 2.** Incidence rates and comparative vaccine effectiveness of HD, adjuvanted, and SD cell-based influenza vaccines in preventing influenza-related outcomes, compared to SD egg-based influenza vaccine, among influenza vaccine recipients ≥65 years of age, stratified by age groups 5](#_Toc168923702)

[**REFERENCES** 6](#_Toc168923703)

# **Supplementary Table 1.** Characteristics of influenza vaccine recipients (≥65 years) by vaccine type (before inverse probability of treatment weighting)

| n (%) | Reference group | Comparison groups | | | ASD |
| --- | --- | --- | --- | --- | --- |
|  | **SD Egg-based**  n=69,006 | **High dose**  n=370,543 | **Adjuvanted**  n=23,508 | **SD Cell-based**  n=32,062 |  |
| **Demographic characteristics** |  |  |  |  |  |
| Age at index date, years |  |  |  |  | 0.255 |
| mean (standard deviation) | 73.30 (6.82) | 74.63 (7.05) | 73.39 (6.83) | 75.08 (7.12) |  |
| median (Q1, Q3) | 72 (68, 77) | 73 (69, 79) | 72 (68, 78) | 74 (69, 79) |  |
| min, max | 65, 104 | 65, 110 | 65, 103 | 65, 104 |  |
| Age at index date, years |  |  |  |  | 0.216 |
| 65-74 | 43,647 (63.3) | 206,737 (55.8) | 20,065 (62.6) | 12,378 (52.7) |  |
| ≥75 | 25,359 (36.7) | 163,806 (44.2) | 11,997 (37.4) | 11,130 (47.3) |  |
| Sex |  |  |  |  | 0.105 |
| Female | 38,532 (55.8) | 203,472 (54.9) | 17,940 (56.0) | 11,896 (50.6) |  |
| Male | 30,474 (44.2) | 167,071 (45.1) | 14,122 (44.0) | 11,612 (49.4) |  |
| Race/Ethnicity |  |  |  |  | 0.155 |
| Non-Hispanic White | 29,807 (43.2) | 173,890 (46.9) | 13,560 (42.3) | 11,846 (50.4) |  |
| Non-Hispanic Black | 6,993 (10.1) | 29,671 (8.0) | 2,359 (7.4) | 2,079 (8.8) |  |
| Hispanic | 20,949 (30.4) | 104,804 (28.3%) | 10,912 (34.0%) | 6,421 (27.3) |  |
| Non-Hispanic Asian | 8,911 (12.9) | 49,927 (13.5) | 4,223 (13.2) | 2,335 (9.9) |  |
| Other/Unknown | 2,346 (3.4) | 12,251 (3.3) | 1,008 (3.1) | 827 (3.5) |  |
| Medicaid | 4,521 (6.6) | 22,117 (6.0) | 2,312 (7.2) | 1,459 (6.2) | 0.026 |
| Neighborhood median household income |  |  |  |  | 0.074 |
| <$40,000 | 1,283 (1.9) | 5,527 (1.5) | 478 (1.5) | 469 (2.0) |  |
| $40,000-$59,999 | 9,285 (13.5) | 44,455 (12.0) | 4,707 (14.7) | 2,901 (12.3) |  |
| $60,000-$79,999 | 14,266 (20.7) | 72,414 (19.5) | 7,312 (22.8) | 5,413 (23.0) |  |
| ≥$80,000 | 43,942 (63.7) | 247,094 (66.7) | 19,460 (60.7) | 14,630 (62.2) |  |
| Unknown | 230 (0.3) | 1,053 (0.3) | 105 (0.3) | 95 (0.4) |  |
| Smoking^a^ |  |  |  |  |  |
| No | 49,525 (71.8) | 265,327 (71.6) | 23,209 (72.4) | 16,251 (69.1) | 0.104 |
| Yes | 17,941 (26.0) | 96,721 (26.1) | 8,262 (25.8) | 6,330 (26.9) |  |
| Unknown | 1,540 (2.2) | 8,495 (2.3) | 591 (1.8) | 927 (3.9) |  |
| **Clinical characteristics** |  |  |  |  |  |
| Body mass index^a^, kg/m^2^ |  |  |  |  | 0.103 |
| <18.5 | 1,026 (1.5) | 6,827 (1.8) | 483 (1.5) | 400 (1.7) |  |
| 18.5 - <25 | 18,371 (26.6) | 105,988 (28.6) | 8,458 (26.4) | 6,379 (27.1) |  |
| 25 - <30 | 24,395 (35.4) | 130,035 (35.1) | 11,385 (35.5) | 8,115 (34.5) |  |
| ≥30 | 22,539 (32.7) | 112,476 (30.4) | 10,702 (33.4) | 7,217 (30.7) |  |
| Unknown | 2,675 (3.9) | 15,217 (4.1) | 1,034 (3.2) | 1,397 (5.9) |  |
| Charlson comorbidity score^b,c^ |  |  |  |  | 0.102 |
| 0 | 22,620 (32.8) | 117,155 (31.6) | 10,236 (31.9) | 6,831 (29.1) |  |
| 1 | 13,995 (20.3) | 75,124 (20.3) | 6,553 (20.4) | 4,471 (19.0) |  |
| ≥2 | 32,391 (46.9) | 178,264 (48.1) | 15,273 (47.6) | 12,206 (51.9) |  |
| Frailty index^b,d^ |  |  |  |  | 0.124 |
| Quartile 1 | 17,240 (25.0) | 93,298 (25.2) | 7,736 (24.1) | 5,478 (23.3) |  |
| Quartile 2 | 17,761 (25.7) | 92,560 (25.0) | 8,102 (25.3) | 5,389 (22.9) |  |
| Quartile 3 | 17,416 (25.2) | 92,349 (24.9) | 8,199 (25.6) | 5,813 (24.7%) |  |
| Quartile 4, most frail | 16,589 (24.0) | 92,336 (24.9) | 8,025 (25.0) | 6,828 (29.0) |  |
| Chronic diseases^b^ |  |  |  |  |  |
| Kidney disease | 12,754 (18.5) | 73,386 (19.8) | 5,927 (18.5) | 5,199 (22.1) | 0.090 |
| Heart disease | 6,649 (9.6) | 38,019 (10.3) | 3,026 (9.4) | 2,881 (12.3) | 0.084 |
| Liver disease | 3,717 (5.4) | 18,422 (5.0) | 1,738 (5.4) | 1,300 (5.5) | 0.019 |
| Diabetes | 22,269 (32.3) | 116,660 (31.5) | 10,772 (33.6) | 7,608 (32.4) | 0.028 |
| Immunocompromised | 3,896 (5.6) | 20,404 (5.5) | 1,730 (5.4) | 1,592 (6.8) | 0.047 |
| Respiratory conditions^b^ |  |  |  |  |  |
| Chronic obstructive pulmonary disease, chronic bronchitis, or emphysema | 5,161 (7.5) | 29,599 (8.0) | 2,336 (7.3) | 2,132 (9.1) | 0.058 |
| Asthma | 6,788 (9.8) | 35,312 (9.5) | 3,231 (10.1) | 2,456 (10.4) | 0.020 |
| **Healthcare utilization** |  |  |  |  |  |
| Number of outpatient and virtual visits^b^ |  |  |  |  | 0.158 |
| 0 | 1,116 (1.6) | 5,786 (1.6) | 395 (1.2) | 880 (3.7) |  |
| 1-4 | 9,499 (13.8) | 55,206 (14.9) | 4,292 (13.4) | 2,807 (11.9) |  |
| 5-10 | 20,642 (29.9) | 116,109 (31.3) | 9,460 (29.5) | 6,263 (26.6) |  |
| ≥11 | 37,749 (54.7) | 193,442 (52.2) | 17,915 (55.9) | 13,558 (57.7) |  |
| Number of Emergency Department visits^b^ |  |  |  |  | 0.066 |
| 0 | 53,311 (77.3) | 286,269 (77.3) | 24,658 (76.9) | 17,560 (74.7) |  |
| 1 | 10,367 (15.0) | 55,008 (14.8) | 4,864 (15.2) | 3,769 (16.0) |  |
| ≥2 | 5,328 (7.7) | 29,266 (7.9) | 2,540 (7.9) | 2,179 (9.3) |  |
| Number of hospitalizations^b^ |  |  |  |  | 0.066 |
| 0 | 63,685 (92.3) | 340,599 (91.9) | 29,672 (92.5) | 21,299 (90.6) |  |
| 1 | 3,905 (5.7) | 21,373 (5.8) | 1,790 (5.6) | 1,525 (6.5) |  |
| ≥2 | 1,416 (2.1) | 8,571 (2.3) | 600 (1.9) | 684 (2.9) |  |
| Preventive care^b^ | 39,746 (57.6) | 20,6141 (55.6) | 18,616 (58.1) | 12,835 (54.6) | 0.061 |
| History of receipt of influenza vaccine^e^ | 62,644 (90.8) | 341,568 (92.2) | 29,218 (91.1) | 21,897 (93.1) | 0.087 |
| Receipt of COVID-19 vaccine^b^ | 59,466 (86.2) | 324,616 (87.6) | 27,694 (86.4) | 21,245 (90.4) | 0.131 |
| Concomitant vaccines^f^ | 16,568 (24.0) | 84,067 (22.7) | 7,716 (24.1) | 4,915 (20.9) | 0.074 |
| Month of index vaccination |  |  |  |  | 0.463 |
| August 2022 | 5,275 (7.6) | 17,979 (4.9) | 3,586 (11.2) | 2,154 (9.2) |  |
| September 2022 | 39,271 (56.9) | 168,827 (45.6) | 17,355 (54.1) | 17,622 (75.0) |  |
| October 2022 | 14,365 (20.8) | 121,162 (32.7) | 6,970 (21.7) | 2,377 (10.1) |  |
| November 2022 | 6,249 (9.1) | 44,990 (12.1) | 2,970 (9.3) | 887 (3.8) |  |
| December 2022 | 3,846 (5.6) | 17,585 (4.7) | 1,181 (3.7) | 468 (2.0) |  |

ASD=absolute standardized difference; SD=standard dose; Q = quartile

^a^ Defined in the two years prior to index date

^b^ Defined in the one year prior to index date

^c^ Possible range: 0-29 ^1^

^d^ Possible range: 0-1 ^2^

^e^ During previous influenza season (August 2021– April 2022)

^f^ Among individuals who received concomitant vaccines received with the index influenza vaccine: COVID-19 vaccine (40.9%); shingles vaccine (47.9%); pneumococcal vaccine (12.1%); Tdap (9.0%); and other vaccines (0.7%)

# **Supplementary Table 2.** Incidence rates and comparative vaccine effectiveness of HD, adjuvanted, and SD cell-based influenza vaccines in preventing influenza-related outcomes, compared to SD egg-based influenza vaccine, among influenza vaccine recipients ≥65 years of age, stratified by age groups

|  | Reference group | | | Comparison groups | | | | | | | | | | | |
| --- | --- | --- | --- | --- | --- | --- | --- | --- | --- | --- | --- | --- | --- | --- | --- |
| **65 – 74 years** | **SD Egg-based (n=43,647)** | | | **HD (n=206,737)** | | | | **Adjuvanted (n =12,378)** | | | | **SD Cell-based (n=20,065)** | | | |
| Outcomes | no. cases | no. p-y | Incidence/1,000 p-y  (95% CI) | no. cases | no. p-y | Incidence/1,000 p-y  (95% CI) | Adjusted^a^ cVE (%) (95% CI) | no. cases | no. p-y | Incidence/1,000 p-y  (95% CI) | Adjusted cVE (%) (95% CI) | no. cases | no. p-y | Incidence/1,000 p-y  (95% CI) | Adjusted^a^ cVE (%) (95% CI) |
| Influenza-related medical encounters^c^ | 386 | 25,269.6 | 15.28 (13.82, 16.88) | 1,611 | 116,852.6 | 13.79 (13.13, 14.48) | 6.2 (-4.7, 16.2) | 101 | 7,573.2 | 13.34 (10.97, 16.21) | 16.4 (-4.9, 33.5)^a^ | 195 | 11,755.5 | 16.59 (14.42, 19.09) | -4.8 (-20.3, -12.1) |
| PCR-confirmed hospitalization^d^ | 21 | 25,410.2 | 0.83 (0.54, 1.27) | 65 | 117,443.1 | 0.55 (0.43, 0.71) | 32.5 (-9.6, 58.9) | 2 | 7,610.8 | 0.26 (0.07, 1.05) | 80.1 (-23.2, 97.0)^b^ | 8 | 11,830.9 | 0.68 (0.34, 1.35) | 16.6 (-47.2, 63.3) |
| ≥**75 years** | **SD Egg-based (n=25,359)** | | | **HD (n=163,806)** | | | | **Adjuvanted (n =11,130)** | | | | **SD Cell-based (n=11,997)** | | | |
| Outcomes | no. cases | no. p-y | Incidence/1,000 p-y  (95% CI) | no. cases | no. p-y | Incidence/1,000 p-y  (95% CI) | Adjusted^e^ cVE (%) (95% CI) | no. cases | no. p-y | Incidence/1,000 p-y  (95% CI) | Adjusted^e^ cVE (%) (95% CI) | no. cases | no. p-y | Incidence/1,000 p-y  (95% CI) | Adjusted^e^ cVE (%) (95% CI) |
| Influenza-related medical encounters^c^ | 239 | 14,917.9 | 16.02 (14.11, 18.19) | 1,321 | 93212.5 | 14.17 (13.43, 14.96) | 12.3 (-0.7, 23.5) | 100 | 6,723.7 | 14.87 (12.23, 18.09) | 18.8 (-4.2, 36.9) | 135 | 7119.6 | 18.96 (16.02, 22.45) | -8.0 (-26.0, 12.6) |
| PCR-confirmed hospitalization^d^ | 32 | 14,999.2 | 2.13 (1.51, 3.02) | 169 | 93660.6 | 1.80 (1.55, 2.10) | 21.3 (-12.2, 45.6) | 7 | 6,762.0 | 1.04 (0.49, 2.17) | 54.6 (-4.0, 80.3) | 12 | 7168.3 | 1.67 (0.95, 2.95) | 30.7 (-26.8, 64.9) |

.

Abbreviations: CI=confidence interval; cVE=comparative vaccine effectiveness; HD=high dose; no. = number; p-y= person-years; PCR = polymerase chain reaction; SD=standard dose

When the hazard ratio or its 95% CI was >1, the cVE (%) or its 95% CI was transformed as ([1/hazard ratio] – 1) × 100

^a^ Weighted using stabilized inverse probability of treatment weights, and additionally adjusted for age and index month

^b^ Weighted using stabilized inverse probability of treatment weights. Hospitalization model was not further adjusted for age and index month due to convergence issues

^c^ A positive influenza PCR test; or influenza-related medical encounter in the outpatient, inpatient, emergency, or virtual visit setting identified based on the U.S. Armed Forces Health Surveillance Center Code Set B^3^ for influenza without an influenza PCR-negative test ±3 days of the code date

^d^ PCR-confirmed influenza-related hospitalization (a positive PCR test collected between -14 and +3 days from the inpatient admission date) with an acute respiratory infection code^4^

^e^ Weighted using stabilized inverse probability of treatment weights, and additionally adjusted for outpatient and virtual visits

#

# **REFERENCES**

1. Quan H, Li B, Couris CM, et al. Updating and validating the Charlson comorbidity index and score for risk adjustment in hospital discharge abstracts using data from 6 countries. *American journal of epidemiology*. Mar 15 2011;173(6):676-82. doi:10.1093/aje/kwq433

2. Kim DH, Schneeweiss S, Glynn RJ, Lipsitz LA, Rockwood K, Avorn J. Measuring Frailty in Medicare Data: Development and Validation of a Claims-Based Frailty Index. *J Gerontol A Biol Sci Med Sci*. Jun 14 2018;73(7):980-987. doi:10.1093/gerona/glx229

3. Armed Forces Health Surveillance Center. INFLUENZA-LIKE ILLNESS (ILI) Code Sets: Code Set B. Updated 2015 October. Accessed 2023 July 18, <https://www.health.mil/Reference-Center/Publications/2015/10/01/Influenza-Like-Illness>

4. Tenforde MW, Weber ZA, DeSilva MB, et al. Vaccine Effectiveness Against Influenza-Associated Urgent Care, Emergency Department, and Hospital Encounters During the 2021-2022 Season, VISION Network. *J Infect Dis*. Jul 14 2023;228(2):185-195. doi:10.1093/infdis/jiad015
